# Active Yeast but Not Henhouse Environment Affects Dropping Moisture Levels in Egg-Laying Hens

**DOI:** 10.3390/ani11082179

**Published:** 2021-07-23

**Authors:** Xue Cheng, Yuchen Liu, Zhong Wang, Lujiang Qu, Zhonghua Ning

**Affiliations:** 1 National Engineering Laboratory for Animal Breeding, Key Laboratory of Animal Genetics, Breeding and Reproduction, Ministry of Agriculture and Rural Affairs, College of Animal Science and Technology, China Agricultural University, Beijing 100193, China; chengxue@cau.edu.cn (X.C.); cauliuyuchen@163.com (Y.L.); 2 State Key Laboratory of Animal Nutrition, College of Animal Science and Technology, China Agricultural University, Beijing 100193, China; wangzh@cau.edu.cn

**Keywords:** egg layers, dropping moisture, feed additives, production performance, egg quality, yeast supplementation, chicken feces

## Abstract

**Simple Summary:**

The high dropping moisture content of chicken feces can impose a serious burden on poultry production costs and the environment. In the first part of this study, we investigated the correlations among chicken dropping moisture content, environmental factors, and production performance. In the second part, we explored whether the addition of three types of additives added individually could reduce the dropping moisture content. The results showed that the dropping moisture level was not associated with production performance or any environmental factors at different locations at the same henhouse height. The probiotic additive (active yeast) significantly reduced the dropping moisture rate. These findings can improve strategies for dealing with high dropping moisture levels and contribute to the enhancement of chicken production.

**Abstract:**

Dropping moisture (DM) refers to the water content in feces. High DM negatively affects poultry production, environment, production costs, and animal health. Heredity, nutrition, environment, and disease may affect DM level. DM has medium inheritability and is related to cage height in henhouses. We examined the relationship among DM level, production performance, and environmental factors at different locations at the same henhouse height and effects of three types of additives. We measured the correlation between environmental factors including temperature, humidity, CO_2_ concentration, absolute pressure, and DM levels and laying performance of 934 Rhode Island Red hens. DM level was not significantly associated with environmental factors or production performance. We divided 64 persistently high DM hens into control and treatment groups supplied with different additives (probiotics, anisodamine, and antibiotics). DM levels, laying performance, egg quality, and serum biochemical indices were determined. Compared with the control and antibiotics, probiotics significantly reduced DM levels and eggshell strength while improving yolk color but did not significantly affect production performance. The additives reduced the b value of eggshell color; compared with probiotics, anisodamine decreased serum globulin levels. Exogenous active yeast supplementation can significantly reduce DM levels.

## 1. Introduction

Dropping moisture (DM) commonly refers to the water content in feces, and DM content is generally higher in poultry than in other animals because of their short digestive tract and the mixture of feces and urine excreted through the cloaca [1]. Furthermore, high DM content in feces exerts adverse effects on the health and economic benefits of laying hens. High DM content can be attributed to pathogenic and non-pathogenic elements. Pathogenic high DM can be caused by infections due to viruses (such as astrovirus, coronaviruses, and Marek’s virus) [2,3,4], bacteria (such as *Escherichia coli*) [5], or fungi (such as *Candida* sp. and *Aspergillus* sp.) [6]. Non-pathogenic high DM is usually related to genetics [1] or changes in the environment or diet [7,8].

As live microorganisms, probiotics are considered to exert beneficial effects on the host [9]. Studies showed that yeast supplementation can enhance the immune functions of breeder laying hens [10], improve intestinal digestive enzyme activities, and improve the performance of aged layers [11]. In addition, there is evidence that the administration of yeast can protect humans and animals from diarrhea prophylactically and therapeutically [12,13]. Anisodamine is a non-specific cholinergic antagonist that inhibits gland secretion. It can effectively relieve pain and vasospasm, improve blood circulation, and block the M receptor, thus suppressing the movement of gastrointestinal (GI) smooth muscles; this prolongs the residence time of chyme in the intestinal tract and improves water reabsorption efficiency [14]. Oregano phenol is an essential oil extracted from oregano plants and is mainly composed of thymol and carvacrol. It has been well documented to have bacteriostatic activities against some Gram-negative and Gram-positive bacteria [15] as well as antioxidant properties [16]. Owing to their characteristics, these additives can be beneficial in promoting production performance and animal health [17,18].

Our previous study showed that DM is a medium-inheritable trait and DM level is related to cage height in henhouses [1]. Therefore, we speculated whether there were differences in DM levels at different locations at the same henhouse cage height. To further explore the mechanism underlying high DM content, we detected the correlation among DM level, environmental factors at different locations at the same henhouse height, and production performance and expected to alleviate this high DM state effectively via treatment with different additives.

## 2. Materials and Methods

This study was approved by the Animal Care and Use Committee of China Agricultural University (permit number: AW30601202-1-1).

### 2.1. DM Level Phenotyping

To exclude high DM content caused by common infections due to *Salmonella* Pullorum (SP) and avian leukosis virus (ALV), SP antibody and ALV p27 antigen titers were obtained to ensure that the hens were free of infection by these pathogens. DM levels in feces were graded according to a previously published study [1]. Briefly, the DM level was identified by appearance and subjectively divided into four grades (1–4), each corresponding to the water content of the feces (i.e., normal, slight, medium, and severe, respectively). In this study, DM levels 3–4 were defined as high DM and levels 1–2 were defined as low DM (normal water content).

### 2.2. Hens, Feed, and Management

A total of 934 Rhode Island Red hens (age: 30 weeks) were raised in the third level of H-type cages. The cage density was one hen per cage. The henhouse was 71-m long, 10.5-m wide, and 2.8-m high with a north-south orientation. Feed and water were provided ad libitum, with a photoperiod of 16 h:8 h (light:dark).

We then selected 64 Rhode Island Red hens on the same tier with high DM levels (levels 3 and 4) by 7 d pre-recording and randomly divided them into four groups of 16 hens each which were then housed individually in single cages, thus resulting in 16 replicate per treatment. Except for the control group (administered only water), we treated the other three groups with different additives, including anisodamine (water with 2.5 mg/mL anisodamine), probiotics (water with 0.05 g/mL probiotics), and antibiotics (water with 0.0125 mL/mL fungicide). After 7 d of adaptation, all hens were treated with 1 mL of each of the three additives by oral gavage at 9:00 a.m. and 2:00 p.m. for the next 14 d at a layer breeding company (Hebei, China). No exogenous antibiotics were administered during the entire trial period. The basal diets mainly comprised maize and soybean meal, and formulated in accordance with the nutrient requirements of laying hens of China (NY/T33-2004) (Table 1). The main ingredient of probiotics (ZHUHAI WANFUKANG BIOTECHNOLOGY Co. Ltd. Guangdong, China) was an active dry yeast at ≥2 billion/g. One liter of fungicide (HEBEI GUANTONG BIOLOGY Co. Ltd. Hebei, China) was composed of 1000 mg oregano phenol, 10000 U glucose oxidase, and 500 mg copper sulfate. Anisodamine was obtained from HENAN RUIPUZHIYAO Co. Ltd. Henan, China.

### 2.3. Sample Collection and Measurements

#### 2.3.1. Environmental Indicators

**Figure 1:**
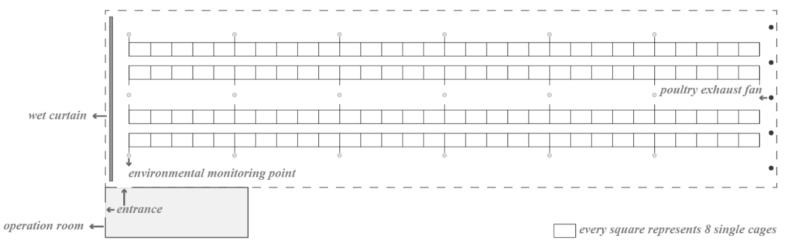
Schematic diagram of environmental indicators test points. The hollow dots represent test points.

In the henhouse, there were four rows with six test sites each, and five groups of 40 hens were housed between two test sites (1.2-m above the ground). The east side of the henhouse was equipped with five exhaust fans and provided with negative-pressure ventilation (Figure 1).

A Testo 480 multi-function measuring instrument (BEIJING AOUDE EQUIPMENT Co. Ltd. Beijing, China) was used to measure environmental indicators, including temperature, humidity, CO_2_ level, and absolute pressure. The measurements were obtained at 3:00 p.m., and the value was recorded after approximately 5 s. Environmental monitoring was performed once a week for a total of three times, two times at each test site, and the average value was taken as the environmental indicator of the test site. At the same time, the DM level between each of the two monitoring points was determined (normal DM was recorded as 0; high DM as 1), and the correlation between environmental indicators of each test site and the corresponding DM level was calculated.

#### 2.3.2. Production Performance

Because hens had a high DM level before starting production and the start production indicators of hens (age, body weight, and weight of the first egg) can have a significant impact on the subsequent production performance, we collected their starting production indicators. Production performance (average egg-laying rate) was evaluated, and qualified eggs during the formal trial were collected. Spearman’s correlation was calculated with DM levels of all 934 Rhode Island Red hens.

The number of eggs laid, abnormal and broken eggs, and double yolk eggs was recorded, and the DM level of each layer was recorded daily for the selected 64 high DM hens that were treated with different additives. Hens were weighed at the beginning and end of the additive treatments.

#### 2.3.3. Egg Quality

For the 64 hens, on days 19–21, 10 eggs per treatment were collected daily to measure egg quality traits. Egg weight was measured using an electronic scale, and the egg index (length/breath) was measured using an egg-shaped index tester. Eggshell color (L*, a*, b*—L* represents brightness, a* includes colors ranging from dark green to bright pink, b* goes from blue to yellow) was measured using an eggshell color tester (Konicaminolta CM-2600d, Tokyo, Japan), and eggshell strength was measured using a quasi-static compression device (Robotmation, Tokyo, Japan). Finally, albumen height, yolk color, and Haugh units were measured using an automatic egg quality analysis instrument (Robotmation EMT-5200, Tokyo, Japan).

#### 2.3.4. Blood Biochemical Parameters

At the end of the experiment, eight laying hens per treatment were randomly selected to provide blood samples from the wing vein. Vacutainer blood tubes containing the blood were placed in a slanted position at room temperature for 4 h, and then, the tubes were centrifuged at 3500 rpm for 10 min. The serum was transferred to 2 mL sterile centrifuge tubes and stored at −20 °C until blood biochemical analysis. Serum total protein, albumin (ALB), globulin (GLB), albumin/globulin (A/G), interferon γ (IFNγ), potassium (K), sodium (Na), chlorine (Cl), calcium (Ca), magnesium (Mg), and phosphorus (P) levels were measured using commercial kits (Nanjing Jiancheng Bioengineering Institute, Nanjing, China).

### 2.4. Statistical Analysis

All graphs were plotted and data analyses were performed using R software (version 4.0.2) and SPSS software (version 24.0; SPSS Inc., Chicago, IL, USA). Results are expressed as the mean and standard error. One-way analysis of variance was performed when data conformed to homogeneity of variance and normal distribution; otherwise, the nonparametric Wilcoxon rank sum test was performed. Differences were considered significant at *p* < 0.05.

## 3. Results and Discussion

### 3.1. Correlation between DM Level, Rearing Environment, and Production Performance

**Figure 2:**
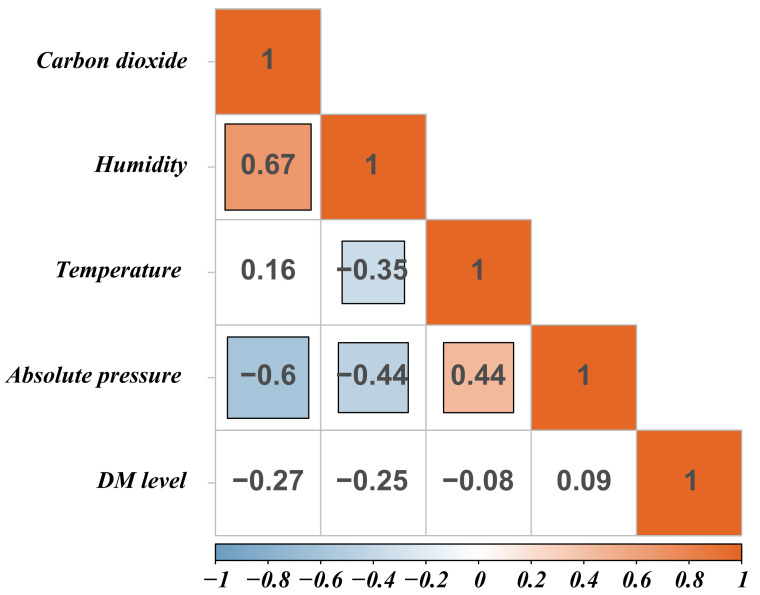
The correlation of high DM level and environmental indicators. Blank background color indicates not significant. The darker the color, the greater the correlation.

In the process of analyzing the environmental indicators at different henhouse locations at the same cage height, we found no significant correlation between DM level and environmental indicators. As expected, humidity was significantly negatively correlated with temperature (r = −0.35) but positively correlated with CO_2_ concentration (r = 0.67). The absolute pressure was significantly negatively correlated with humidity (r = −0.44) and CO_2_ concentration (r = −0.6), whereas it was positively correlated with temperature (r = 0.44) in the henhouse (Figure 2). Previous studies showed that occurrence of seasonal diarrhea can be significantly affected by climatic factors in susceptible populations [19,20]. High temperature increases the probability of diarrhea by prolonging the survival time of bacteria, such as *E. coli* in contaminated food, and changing behavior patterns, such as increasing water consumption and deteriorating sanitary conditions [21], thus leading to increased intestinal permeability and local inflammation [22]. These results indicate that temperature and humidity can affect DM levels. However, our results showed that there was no significant correlation between high DM level and environmental indicators, suggesting that environmental differences at different henhouse locations at the same cage height were not sufficient to cause differences in the distribution of DM.

**Figure 3:**
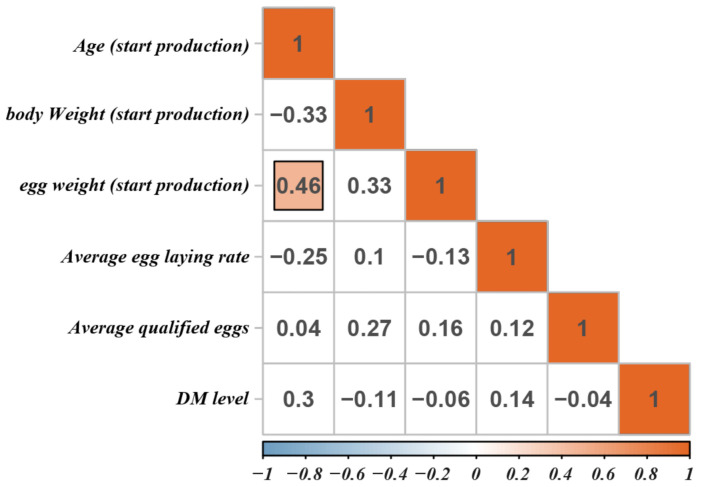
The correlation of high DM level and production performance. Blank background color indicates not significant. The darker the color, the greater the correlation.

The relationship between DM level and production performance is presented in Figure 3. In our study, only the correlation coefficient between the first egg weight and the hen’s age at the first egg was significant (r = 0.46), whereas all others were not significant. High DM level is usually considered to be caused by bacterial and viral infections and is closely related to reduced production performance and depression [23]. Wang et al. [24] found that high DM level caused by *Salmonella* infection for 4 weeks exerted adverse effects on egg production, feed intake, and feed efficiency in laying hens. Harmful bacteria can also attach and colonize the intestinal mucosa, produce toxins that directly affect intestinal health, and negatively affect the performance of the host [25]. Furthermore, *E. coli* causes high DM levels in chickens, increased diet consumption, weight loss, and increased mortality, leading to serious damage to the poultry industry [26]. Previous research showed that common high DM levels can exert adverse effects on animal production performance, organismal health, and mental states. However, in the current study, we found that high DM level was not significantly associated with indexes related to egg production or laying performance. Therefore, we speculated that this high DM level in layers is caused by the insufficiency of water absorption by the epithelial cells of the intestine in the hindgut and did not affect the digestion and absorption of feed nutrients in the foregut.

### 3.2. Effects of Treatment with Different Additives on High DM Levels

To investigate the factors that may contribute to high DM levels, layers with high DM levels within 7 d were included in this experiment, and three different additives were administered to explore the factors that may affect high DM levels. The results showed that compared with the control and antibiotics, probiotics significantly reduced the high DM level (Table 2; *p* < 0.05); however, no significant differences in indexes of laying rate, qualified-egg rate, and body weight were observed among the different groups. Numerous studies showed that yeast can improve the richness and evenness of intestinal microbiota and promote production performance and gut health [27,28]. Several previous studies demonstrated that yeast can improve the abundance of *Lactobacillus* in the gut, which can improve the level of intestinal mucosal immunity and reduce intestinal inflammatory response by regulating the intestinal flora, thereby alleviating DM symptoms [29,30,31]. As reported previously, anisodamine has been widely used to relieve intestinal, microvascular, and airway smooth muscle spasms and effectively inhibit smooth muscle contractility, GI and sweat secretion [14]. When anisodamine was administered alone, it was shown to inhibit intestinal propulsion and minimize diarrhea [32]. However, the results of this experiment are inconsistent with previous findings. Anisodamine did not show a statistically significant effect on relieving high DM levels in the layers, suggesting that high DM level was not associated with excessive gut peristalsis. The prolonged residence time of chyme through the intestinal tract did not increase water absorption, suggesting that the intestinal epithelial cells of hens may be dysfunctional, thereby reducing water absorption capacity, which can result in a high DM level. Thymol nanoemulsion, which is the main active ingredient of oregano phenol, has anti-bacterial and anti-inflammatory efficacy and enhances the stability and absorbability of the GI tract [33]. It has been reported that thymol nanoemulsion supplementation to the diet of *Salmonella*-infected broilers can enhance growth performance, improve the microbial composition of the cecum, and reduce *Salmonella* damage to the cecum and liver tissue [34]. Nevertheless, in our study, we did not observe a decrease in high DM levels and improved production performance by adding thymol nanoemulsion, which indicated that high DM may not be relevant to pathogen infection.

In parallel, egg quality traits were tested, and the results showed that probiotics significantly enhanced yolk color (Table 3; *p* < 0.05). On one hand, fat-soluble pigments in the egg yolk mainly come from diets, such as corn and corn gluten meal, and are absorbed through the intestinal tract and eventually deposited into the egg yolk [35]. Previous studies demonstrated that probiotics can increase the nutrient absorption and utilization by promoting the growth of non-pathogenic facultative anaerobic bacteria while inhibiting the proliferation of intestinal pathogens such as *E. coli* and *Salmonella* [36,37],thereby improving lutein deposition in the egg yolk. On the other hand, yeast fermentation products are rich in carotenoids, which can contribute to the enhancement of yolk color [38]. Interestingly, compared with the other groups, the probiotics group showed decreased eggshell strength (*p* < 0.05). In previous studies, yeast was usually added to the feed, which the chickens consumed and digested slowly [11,29]. However, in this study, yeast was dissolved in water and administered by gavage at two fixed times on each day. This may have resulted in a competitive absorption relationship between the minerals contained in yeast and dietary calcium, leading to a decrease in eggshell strength.

Serum biochemical indices can reflect changes in tissue cell permeability and metabolic function of the body, which is a sensitive index reflecting the state of the animals’ health [39]. In this study, we found that compared with the probiotics group, the anisodamine group showed a decrease in the levels of GLB, and the remaining indexes did not present significant differences (Table 4). Similar to the findings of previous reports, yeast can significantly increase the level of serum GLB [40,41]. In contrast, Patane and Premavalli [42] found that adding different levels of yeast did not affect serum GLB level, which is possibly because of the different breeds of chickens and feeding approach. Additionally, serum GLB levels are associated with the immune ability of the body. The A/G ratio is significantly associated with chronic inflammation [43]. Generally, high DM levels occur as a result of an imbalance in the absorption and secretion of ions and solutes in intestinal epithelial cells, accompanied by altered water transport [44]. Inflammatory DM can trigger the production of immune cell products, including IFNγ, which regulates ion transporters. Ca^2+^ and cyclic AMP can drive Cl^−^ secretion, and water moves into the lumen, resulting in high DM levels [45]. William et al. [46] reported that DM caused by bacterial infection can activate cellular Cl^−^ channels and inhibit Na^+^/H^+^ exchanger 3, leading to a decrease in the absorption of Cl^−^ and Na^+^. However, in the current study, we found that IFNγ and other ions such as Cl^−^, Ca^2+^, and Na^+^ in the serum exhibited no significant difference among the groups. This may be because electrolyte imbalances in the hindgut segment were not present in the serum. Further investigations are warranted on electrolyte and ion metabolism in specific intestinal segments to further elucidate the complex causes of high DM levels.

## 4. Conclusions

High DM levels were not significantly associated with production performance and environmental factors at different locations in the same cage height. Antibacterial agents and anisodamine did not significantly reduce the DM levels. Probiotics can significantly alleviate high DM levels in breeder layers; however, the specific mechanism remains to be further explored.

## Figures and Tables

**Table 1:** Ingredients and nutrient composition of basal diet.

Ingredients	Percent	Nutrient Level ^b^	Percent
Corn (CP 8.3%)	64.0	ME (MJ/Kg)	16.01
Soybean meal (CP 44.0%)	19.8	CP (%)	16.04
Soybean oil	0.7	CF (%)	3.24
Wheat bran	3.0	Methionine (%)	0.24
Limestone	9.5	Lysine (%)	0.70
Calcium hydrogen phosphate	1.00	Calcium (%)	3.49
Sodium chloride	0.30	Total P (%)	0.32
DL-Methionine (98%)	0.10		
L-Lysine HCL (78%)	0.07		
Vitamin premix ^a^	0.03		
Mineral premix ^b^	0.20		
Choline chloride (50%)	0.15		
Phytase	0.02		
NSP enzyme	0.02		
Total	100.0		

Note: ^a^ Supplied per kilogram of diet: vitamin A, 13,500 IU; vitamin D3, 4500 IU; vitamin E, 75 IU; vitamin K3, 3.6 mg; vitamin B1, 3.0 mg; vitamin B2, 9.24 mg; vitamin B6, 6.0 mg; nicotinic acid, 66 mg; pantothenic acid, 16.8 mg; biotin, 0.54 mg; folic acid, 2.10 mg; vitamin B12, 0.03 mg; vitamin C, 135 mg; choline, 675 mg; ethoxyquinoline, 15 mg; ^b^ Mineral premix provided per kilogram of complete diet: iron, 80 mg; copper, 10 mg; manganese, 100 mg; zinc, 100 mg; iodine, 0.35 mg; selenium, 0.30 mg; ^b^ ME, CP, and CF were measured values, and the other nutrients were calculated values.

**Table 2:** The effects of different additives on the production performance and DM level of layers.

	Egg-Laying Rate (%)	Qualified Egg Rate (%)	Initial Body Weight (g)	Final Body Weight (g)	High DM Level (%)
Control group	97.27	99.70	2073.87	2060.67	0.95 ^b^
Anisodamine group	95.76	100.00	2068.93	2067.33	0.86 ^a,b^
Probiotics group	95.13	99.32	2056.40	2036.27	0.85 ^a^
Antibacterial group	96.97	99.08	2062.27	2062.20	0.93 ^b^
SEM	0.57	0.22	13.47	16.09	0.02
*p* Value	0.65	0.28	0.97	0.91	0.03

Note: ^a,b^ Means traits across a column with different superscripts are significantly different (*p* < 0.05).

**Table 3:** Effects of different additives on egg quality of high DM layers.

	Egg Weight, g	Shape Index	Eggshell Strength, kg/cm^3^	Albumen Height, mm	Yolk Color	Haugh Units	L*	a*	b*
Control group	58.87	1.27	3.88 ^a^	7.89	7.06 ^b^	89.06	58.82	18.62	34.34 ^a^
Anisodamine group	60.62	1.27	3.77 ^a^	7.90	7.11 ^a,b^	88.87	59.94	18.26	32.81 ^b^
Probiotics group	59.42	1.28	3.33 ^b^	8.08	7.45^a^	90.13	60.84	17.51	32.64 ^b^
Antibacterial group	60.78	1.28	3.79 ^a^	8.15	7.39 ^ac^	90.05	59.62	18.01	32.28 ^b^
SEM	0.368	0.003	0.066	0.073	0.063	0.398	0.315	0.184	0.238
P	0.18	0.51	0.03	0.61	0.03	0.76	0.36	0.28	<0.01

Note: ^a,b^ Means traits across a column with different superscripts are significantly different (*p* < 0.05).

**Table 4:** Effects of different additives on serum biochemical parameters of high DM layers.

Item	Control Group	Anisodamine Group	Probiotics Group	Antibacterial Group	SEM	*p*-Value
TP (g/L)	48.99	41.84	49.26	45.05	1.29	0.126
ALB (g/L)	20.16	17.85	20.46	19.21	0.44	0.152
GLB (g/L)	28.83	23.99	29.34	25.84	0.87	0.091
A/G	0.71	0.75	0.71	0.75	0.01	0.290
IFNγ (pg/mL)	13.76	25.09	11.98	16.91	2.95	0.423
K (mmol/L)	7.38	7.50	8.23	7.84	0.21	0.481
Na (mmol/L)	140.23	140.38	140.91	140.24	0.14	0.282
Cl (mmol/L)	114.11	108.91	115.44	110.39	1.10	0.115
Ca (mmol/L)	4.36	3.88	4.47	4.20	0.10	0.160
Mg (mmol/L)	1.10	1.04	1.14	1.07	0.03	0.573
P (mmol/L)	1.68	1.54	1.66	1.55	0.06	0.797

## Data Availability

The study did not report any data.

## References

[1] Zhu T., Zhang T.Y., Wen J., Zhao X., Chen Y., Jia Y., Wang L., Lv X., Yang W., Guan Z. (2020). The Genetic Architecture of the Chickens Dropping Moisture by Genetic Parameter Estimation and Genome-Wide Association. Front. Genet..

[2] Gerdts V., Zakhartchouk A. (2017). Vaccines for porcine epidemic diarrhea virus and other swine coronaviruses. Vet. Microbiol..

[3] Li G., Yuan S., Yan T., Shan H., Cheng Z. (2020). Identification and characterization of chicken circovirus from commercial broiler chickens in China. Transbound. Emerg. Dis..

[4] Smyth V.J. (2017). A Review of the Strain Diversity and Pathogenesis of Chicken Astrovirus. Viruses.

[5] Tie K., Yuan Y., Yan S., Yu X., Zhang Q., Xu H., Zhang Y., Gu J., Sun C., Lei L. (2018). Isolation and identification of Salmonella pullorum bacteriophage YSP2 and its use as a therapy for chicken diarrhea. Virus Genes.

[6] Manning R.O., Wyatt R.D. (1984). Toxicity of Aspergillus ochraceus contaminated wheat and different chemical forms of ochratoxin A in broiler chicks. Poult. Sci..

[7] de Koning C., Barekatain R., Singh M., Drake K. (2019). Saltbush (*Atriplex nummularia* and *A. amnicola*) as potential plants for free-range layer farms: Consequences for layer performance, egg sensory qualities, and excreta moisture. Poult. Sci..

[8] Lauridsen C. (2019). From oxidative stress to inflammation: Redox balance and immune system. Poult. Sci..

[9] Vieira A.T., Teixeira M.M., Martins F.S. (2013). The role of probiotics and prebiotics in inducing gut immunity. Front. Immunol..

[10] Zhen W., Shao Y., Wu Y., Li L., Pham V.H., Abbas W., Wan Z., Guo Y., Wang Z. (2020). Dietary yeast beta-glucan supplementation improves eggshell color and fertile eggs hatchability as well as enhances immune functions in breeder laying hens. Int. J. Biol. Macromol..

[11] Zhang J.C., Chen P., Zhang C., Khalil M.M., Zhang N.Y., Qi D.S., Wang Y.W., Sun L.H. (2020). Yeast culture promotes the production of aged laying hens by improving intestinal digestive enzyme activities and the intestinal health status. Poult. Sci..

[12] Kambale R.M., Nancy F.I., Ngaboyeka G.A., Kasengi J.B., Bindels L.B., Van der Linden D. (2020). Effects of probiotics and synbiotics on diarrhea in undernourished children: Systematic review with meta-analysis. Clin. Nutr..

[13] Kayasaki F., Okagawa T., Konnai S., Kohara J., Sajiki Y., Watari K., Ganbaatar O., Goto S., Nakamura H., Shimakura H. (2021). Direct evidence of the preventive effect of milk replacer-based probiotic feeding in calves against severe diarrhea. Vet. Microbiol..

[14] Poupko J.M., Baskin S.I., Moore E. (2007). The pharmacological properties of anisodamine. J. Appl. Toxicol..

[15] Marino M., Bersani C., Comi G. (1999). Antimicrobial activity of the essential oils of Thymus vulgaris L. measured using a bioimpedometric method. J. Food Prot..

[16] Marchese A., Orhan I.E., Daglia M., Barbieri R., Di Lorenzo A., Nabavi S.F., Gortzi O., Izadi M., Nabavi S.M. (2016). Antibacterial and antifungal activities of thymol: A brief review of the literature. Food Chem..

[17] Fernandez M.E., Kembro J.M., Ballesteros M.L., Caliva J.M., Marin R.H., Labaque M.C. (2019). Dynamics of thymol dietary supplementation in quail (Coturnix japonica): Linking bioavailability, effects on egg yolk total fatty acids and performance traits. PLoS ONE.

[18] Hashemipour H., Kermanshahi H., Golian A., Khaksar V. (2014). Effects of carboxy methyl cellulose and thymol + carvacrol on performance, digesta viscosity and some blood metabolites of broilers. J. Anim. Physiol. Anim. Nutr..

[19] Zhou X., Zhou Y., Chen R., Ma W., Deng H., Kan H. (2013). High temperature as a risk factor for infectious diarrhea in Shanghai, China. J. Epidemiol..

[20] Emch M., Feldacker C., Islam M.S., Ali M. (2008). Seasonality of cholera from 1974 to 2005: A review of global patterns. Int. J. Health Geogr..

[21] Checkley W., Epstein L.D., Gilman R.H., Cabrera L., Black R.E. (2003). Effects of acute diarrhea on linear growth in Peruvian children. Am. J. Epidemiol..

[22] Quinteiro-Filho W.M., Calefi A.S., Cruz D.S.G., Aloia T.P.A., Zager A., Astolfi-Ferreira C.S., Piantino Ferreira J.A., Sharif S., Palermo-Neto J. (2017). Heat stress decreases expression of the cytokines, avian beta-defensins 4 and 6 and Toll-like receptor 2 in broiler chickens infected with Salmonella Enteritidis. Vet. Immunol. Immunopathol..

[23] Higgins S.E., Torres-Rodriguez A., Vicente J.L., Sartor C.D., Pixley C.M., Nava G.M., Tellez G., Barton J.T., Hargis B.M. (2005). Evaluation of Intervention Strategies for Idiopathic Diarrhea in Commercial Turkey Brooding Houses. J. Appl. Poult. Res..

[24] Wang W.-W., Jia H.-J., Zhang H.-J., Wang J., Lv H.-Y., Wu S.-G., Qi G.-H. (2019). Supplemental Plant Extracts From Flos lonicerae in Combination With Baikal skullcap Attenuate Intestinal Disruption and Modulate Gut Microbiota in Laying Hens Challenged by Salmonella pullorum. Front. Microbiol..

[25] Wang L.C., Zhang T.T., Wen C., Jiang Z.Y., Wang T., Zhou Y.M. (2012). Protective effects of zinc-bearing clinoptilolite on broilers challenged withSalmonella pullorum. Poult. Sci..

[26] Li H., Ma M.L., Xie H.J., Kong J. (2012). Biosafety evaluation of bacteriophages for treatment of diarrhea due to intestinal pathogen Escherichia coli 3-2 infection of chickens. World J. Microbiol. Biotechnol..

[27] Wu C., Yang Z., Song C., Liang C., Li H., Chen W., Lin W., Xie Q. (2018). Effects of dietary yeast nucleotides supplementation on intestinal barrier function, intestinal microbiota, and humoral immunity in specific pathogen-free chickens. Poult. Sci..

[28] Trckova M., Faldyna M., Alexa P., Sramkova Zajacova Z., Gopfert E., Kumprechtova D., Auclair E., D’Inca R. (2014). The effects of live yeast Saccharomyces cerevisiae on postweaning diarrhea, immune response, and growth performance in weaned piglets. J. Anim. Sci..

[29] Liu Y., Cheng X., Zhen W., Zeng D., Qu L., Wang Z., Ning Z. (2021). Yeast Culture Improves Egg Quality and Reproductive Performance of Aged Breeder Layers by Regulating Gut Microbes. Front. Microbiol..

[30] Guarino A., Guandalini S., Lo Vecchio A. (2015). Probiotics for Prevention and Treatment of Diarrhea. J. Clin. Gastroenterol..

[31] Lai H.H., Chiu C.H., Kong M.S., Chang C.J., Chen C.C. (2019). Probiotic Lactobacillus casei: Effective for Managing Childhood Diarrhea by Altering Gut Microbiota and Attenuating Fecal Inflammatory Markers. Nutrients.

[32] Wang H., Lu Y., Chen H.-Z. (2003). Differentiating effects of anisodamine on cognitive amelioration and peripheral muscarinic side effects induced by pilocarpine in mice. Neurosci. Lett..

[33] Salehi B., Mishra A.P., Shukla I., Sharifi-Rad M., Contreras M.D.M., Segura-Carretero A., Fathi H., Nasrabadi N.N., Kobarfard F., Sharifi-Rad J. (2018). Thymol, thyme, and other plant sources: Health and potential uses. Phytother. Res..

[34] Ibrahim D., Abdelfattah-Hassan A., Badawi M., Ismail T.A., Bendary M.M., Abdelaziz A.M., Mosbah R.A., Mohamed D.I., Arisha A.H., El-Hamid M.I.A. (2021). Thymol nanoemulsion promoted broiler chicken’s growth, gastrointestinal barrier and bacterial community and conferred protection against Salmonella Typhimurium. Sci. Rep..

[35] Bailey C.A., Chen B.H. (1989). Chromatographic Analyses of Xanthophylls in Egg Yolks from Laying Hens Fed Turf Bermudagrass(*Cynodon dactylon*) Meal. J. Food Sci..

[36] Ehrmann M.A., Kurzak P., Bauer J., Vogel R.F. (2002). Characterization of lactobacilli towards their use as probiotic adjuncts in poultry. J. Appl. Microbiol..

[37] Fulton R.M., Nersessian B.N., Reed W.M. (2002). Prevention of Salmonella enteritidis infection in commercial ducklings by oral chicken egg-derived antibody alone or in combination with probiotics. Poult. Sci..

[38] Sun J., Li M., Tang Z., Zhang X., Chen J., Sun Z. (2020). Effects of Rhodotorula mucilaginosa fermentation product on the laying performance, egg quality, jejunal mucosal morphology and intestinal microbiota of hens. J. Appl. Microbiol..

[39] Jia Q., Zhang L., Zhang J., Pei F., Zhu S., Sun Q., Duan L. (2019). Fecal Microbiota of Diarrhea-Predominant Irritable Bowel Syndrome Patients Causes Hepatic Inflammation of Germ-Free Rats and Berberine Reverses It Partially. Biomed. Res. Int..

[40] Hussein E., Selim S. (2018). Efficacy of yeast and multi-strain probiotic alone or in combination on growth performance, carcass traits, blood biochemical constituents, and meat quality of broiler chickens. Livest. Sci..

[41] Paryad. A., Mahmoudi M. (2008). Effect of different levels of supplemental yeast (Saccharomyces cerevisiae) on performance, blood constituents and carcass characteristics of broiler chicks. Afr. J. Agric. Res..

[42] Shankar. P.A., Premavalli. K., Omprakash A.V., Kirubakaran J.J., Hudson G.H., Vairamuthu S. (2018). Effect of Dietary Yeast Supplementation on Serum Biochemical Profile of Broiler Chicken. Indian Vet. J..

[43] Delanaye P., Park J., Kim H.J., Kim J., Choi Y.B., Shin Y.S., Lee M.J. (2020). Predictive value of serum albumin-to-globulin ratio for incident chronic kidney disease: A 12-year community-based prospective study. PLoS ONE.

[44] Viswanathan V.K., Hodges K., Hecht G. (2009). Enteric infection meets intestinal function: How bacterial pathogens cause diarrhoea. Nat. Rev. Microbiol..

[45] Gareau M.G., Barrett K.E. (2013). Fluid and electrolyte secretion in the inflamed gut: Novel targets for treatment of inflammation-induced diarrhea. Curr. Opin. Pharmacol..

[46] Petri W.A., Miller M., Binder H.J., Levine M.M., Dillingham R., Guerrant R.L. (2008). Enteric infections, diarrhea, and their impact on function and development. J. Clin. Investig..

